# Data related to crystalline photovoltaic plant performance in the semi-arid climate of India

**DOI:** 10.1016/j.dib.2020.105696

**Published:** 2020-05-19

**Authors:** Nallapaneni Manoj Kumar, Maria Malvoni, Nikos Hatziargyriou, Shauhrat S Chopra

**Affiliations:** a School of Energy and Environment, City University of Hong Kong, Kowloon, Hong Kong; b School of Electrical and Computer Engineering, National Technical University of Athens, Greece

**Keywords:** Photovoltaic performance, Photovoltaic derate, Performance losses, Degradation rate, Semi-arid climates, Solar PV plant in south India

## Abstract

This article presents performance data concerning a 1MW crystalline photovoltaic (PV) plant installed in the semi-arid climate of India. Data includes the daily average samples from January 2012 to February 2016, related to solar irradiance on the plane of the array, electrical energy injected into the grid, reference yield, final yield, and the performance ratio. Furthermore, the decomposition time series for the performance ratio by applying the classical seasonal decomposition (CSD), Holt-Winters seasonal model (HW), and Seasonal and Trend decomposition using Loess (STL) is also provided for quantifying of the degradation rate of the PV system. The data are provided in the supplementary file included in this article. The dataset is related to the paper entitled “Performance and degradation assessment of large-scale grid-connected solar photovoltaic power plant in tropical semi-arid environment of India.” [1].

**Specifications Table**

**Table:** 

**Subject**	Renewable Energy, Sustainability and the Environment
**Specific subject area**	Outdoor performance, energy performance, and degradation analysis of utility-scale photovoltaic systems.
**Type of data**	Table Graph
**How data were acquired**	A central monitoring unit (CMU), along with supervisory control and data acquisition (SCADA) system, record the output of the solar PV plant. A weather data logging unit monitors and records the solar radiation.
**Data format**	Raw Analyzed
**Parameters for data collection**	Fifty months of outdoor performance analysis of a 1 MW grid-connected PV system (January 2012 to February 2016) were computed by using daily samples of the solar irradiance and the energy generation.
**Description of data collection**	The dataset includes the daily average of the solar irradiance on the plane of the array and the electrical energy injected into the grid that were collected by a SCADA on a daily base for four years of outdoor exposure. The Reference Yield, the Final Yield, and the Performance Ratio were evaluated. Statistical methods as CSD, HW, and STL are applied to the time series for the performance ratio to get the corresponding decomposition. Linear regression is applied to each decomposition time series for the performance ratio to investigate the degradation rate.
**Data source location**	The utility-scale solar PV power plant is in Telangana State, India. Latitude 16.3 °N; Longitude 77.7 °E, Altitude: 401 meters.
**Data accessibility**	With the article
**Related research article**	Malvoni M, Kumar NM, Chopra SS, Hatziargyriou N, Performance and degradation assessment of 1MW grid-connected PV power plant in tropical semi-arid environment of India, 2020, Solar Energy, 203:101-113.

**Value of the Data**

• The dataset on the PV generation performance is essential for the feasibility assessment of developing solar PV projects. The shared data can be used to perform simulation studies for a stand-alone and grid-connected photovoltaic power system in a tropical semi-arid climate.
• These data are an important reference source for performance comparison studies of PV systems in different sites and under various operating conditions.
• The performance knowledge is a crucial key to ensure the reliability of the PV systems by fault diagnostic techniques that can be investigated by applied the data here presented.
• The aging and failures of PV plant components cause energy losses during the lifetime; therefore, methods for determining the degradation of PV power plants can be analyzed by using the shared data.

## 1 Data Description

**Fig. 1:**
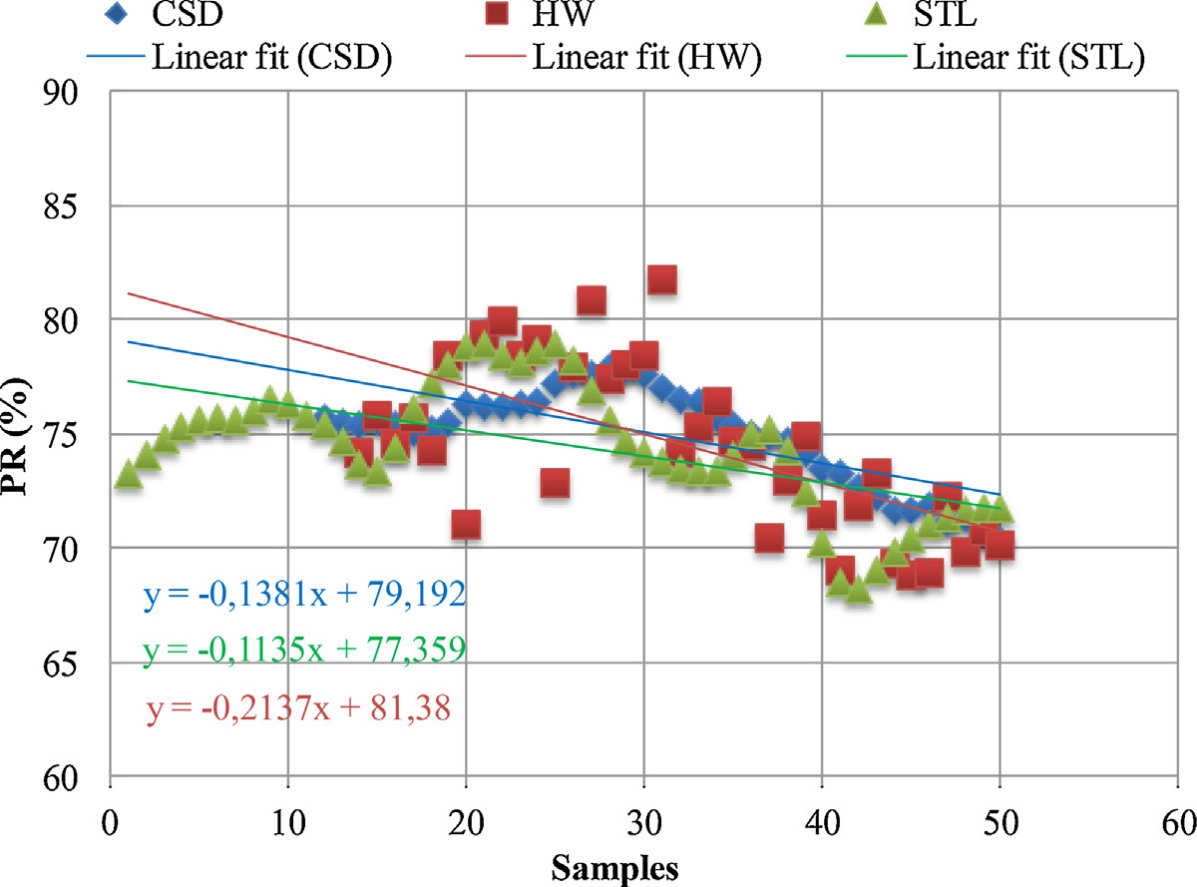
The linear fit for the PR decomposition by the CSD, HW, and STL methods.

The dataset includes the daily average samples of the solar irradiance on the plane of array (G_POA_), the electrical energy injected into the grid (E_AC_), the Reference Yield (Y_R_), the Final Yield (Y_F_) and the Performance Ratio (PR) related to a grid-connected 1 MW_P_ PV system installed in the Telangana State of South India. The data refers to four years of operating conditions from January 2012 to February 2016. The decomposition time series for the performance ratio, by applying the classical seasonal decomposition (CSD), Holt-Winters seasonal model (HW), and seasonal and trend decomposition using loess (STL), is provided. The dataset is provided in the Supplementary material.xlsx. Furthermore, such decompositions are employed to investigate the performance losses by a linear regression model (LR). The LR's coefficients are shown in Fig 1 and applied to quantify the degradation rate of the PV system.

### 1.1 Experimental Design, Materials, and Methods

The data is related to the grid-connected 1 MWp PV system is located in the Telangana State of India (Lat 16.3 °N, Log 77.7 °E, Alt 401 meter) under Tropical semi-arid climate. The utility-scale PV plant consists of 4284 mono-crystalline PV modules (each module of 235 kW_P_ capacity) and 4 DC/AC converter (each converter of 250 KWp capacity). Additional details of the PV system can be found in [1]. A supervisory control and data acquisition (SCADA) system are installed to monitor and record weather data and the output of the solar PV plant that is feeding into the grid.

The data associated with this paper can be found in the Supplementary material.xlsx. The Sheet 1 contains 50 rows related to the daily average of G_POA_ (kWh/m^2^/day), E_AC_ (kWh/day) Y_F_ (h/day), Y_R_ (h/day) and PR (%) recorded from January 2012 to February 2016.

The PV system performance indicators are determined according to the standard IEC 61724 [2] as follows:

(1) $$Y_{R}=\frac{G_{POA}}{G_{STC}\;}\;\;\left[h/day\right]\;\;\;\;\;$$

(2) $$Y_{F}=\frac{E_{AC}}{P_{rated}}\;\;\;\;\left[h/day\right]\;\;\;\;$$

(3) $$PR=\frac{Y_{F}}{Y_{R}}*100\;\;\;\left[\%\right]$$

where *G_STC_* (W/m^2^/day) is reference irradiance at standard test conditions (STC) of 1000 W/m^2^ and *P_rated_* (kW) the installed nominal power.

The PR time series decomposition by using the CSD, HW, and STL methods is reported in Sheet 2 of the Supplementary material.xls file. The dataset consists of 3 columns and 39, 37, and 50 rows, respectively, for each method. More details regarding their implementation are presented in [1].

The degradation rate (DR) is given by [3]:

(4) $$DR=12*\frac{a}{b}*100\;\;\left[\%\right]$$

where ‘a and ‘b’ are the coefficients of the linear regression. Such coefficients are computed for the PR decomposition, according to CSD, HW, and STL methods, and shown in Fig. 1.

## Declaration of Competing Interest

The authors declare that they have no known competing financial interests or personal relationships which have, or could be perceived to have, influenced the work reported in this article.
